# Stability of Root Coverage Outcomes After Soft‐Tissue Augmentation With a Collagen Matrix With or Without rhPDGF‐BB: A 3‐Year Triple‐Blinded, Randomised, Placebo‐Controlled Trial

**DOI:** 10.1111/jcpe.70030

**Published:** 2025-09-11

**Authors:** Lorenzo Tavelli, Shayan Barootchi, Maria Vera Rodriguez, Leonardo Mancini, Hamoun Sabri, Tu Nguyen, Jad Majzoub, Suncica Travan, William V. Giannobile

**Affiliations:** ^1^ Department of Oral Medicine, Infection, and Immunity, Division of Periodontology Harvard School of Dental Medicine Boston Massachusetts USA; ^2^ Center for Clinical Research and Evidence Synthesis in Oral TissuE RegeneratION (CRITERION) Boston Massachusetts USA; ^3^ Department of Periodontics & Oral Medicine University of Michigan School of Dentistry Ann Arbor Michigan USA; ^4^ School of Dentistry Universidad Catolica de Santiago de Guayaquil (UCSG) Guayaquil Ecuador; ^5^ Clinic of Reconstructive Dentistry, Center of Dental Medicine University of Zurich Zurich Switzerland

**Keywords:** collagen matrix, follow‐up study, gingival recession, platelet‐derived growth factor, randomised clinical trial, root coverage, root coverage stability

## Abstract

**Aim:**

To evaluate the 3‐year stability of root coverage outcomes following treatment with a coronally advanced flap (CAF) and a volume‐stable, cross‐linked xenogeneic collagen matrix (VCMX), either soaked in recombinant human platelet–derived growth factor‐BB (rhPDGF, test arm) or sterile saline (control arm).

**Methods:**

Of the original 30 participants in the triple‐blind, randomised, placebo‐controlled trial, 26 were available for follow‐up at 1 and 3 years. The primary outcome was mean root coverage (mRC) at 3 years compared to the 6‐month results. Secondary outcomes included complete root coverage, keratinised tissue width, gingival thickness, volumetric and aesthetic changes, patient‐reported outcome measures (PROMs) and ultrasonographic tissue properties. Untreated contralateral teeth were also assessed.

**Results:**

From 6 months to 3 years, mRC decreased by 6.7% in the control arm and 7.5% in the test arm (*p* > 0.05). Over 90% of treated sites maintained a stable gingival margin (≤ 0.5 mm shift). No significant inter‐group differences were found for volumetric, aesthetic or PROM outcomes. Ultrasonography revealed increased tissue elasticity in treated sites. In contrast, untreated contralateral sites showed progressive gingival recession and increased hypersensitivity (*p* < 0.001).

**Conclusion:**

Both VCMX treatments demonstrated stable clinical and ultrasonographic outcomes over 3 years, while untreated sites showed signs of soft‐tissue deterioration.

## 1 Introduction

Root coverage procedures with autogenous connective tissue graft (CTG) are effective for treating gingival recessions (GRs) (Chambrone et al. 2022; Zucchelli et al. 2020). However, for large surgical sites with multiple adjacent gingival recessions (MAGRs), CTG presents limitations, such as increased surgical time, intra‐operative palatal bleeding and greater postoperative morbidity (Tavelli, Barootchi, Stefanini, et al. 2023). A negative palatal harvesting experience can also affect patients' willingness to undergo future surgeries (Tavelli et al. 2021). As a result, soft‐tissue graft substitutes have been widely explored (Aroca et al. 2013; Cairo 2017; Jepsen et al. 2017; Moslemi et al. 2011). These biomaterials offer advantages including no donor site morbidity and unlimited availability, making them appealing for MAGRs (McGuire et al. 2022; McGuire et al. 2014; Tavelli et al. 2020b).

A novel xenogeneic, porous, cross‐linked, volume‐stable collagen matrix (VCMX) was recently introduced for soft‐tissue augmentation (Stefanini et al. 2020; Thoma et al. 2017; Thoma et al. 2016). VCMX is chemically cross‐linked for enhanced mechanical stability (Asparuhova et al. 2021; Mathes et al. 2010). Its properties make it suitable both as a graft substitute and as a carrier scaffold (Agis et al. 2014; Barootchi et al. 2022). An ex vivo study showed enhanced cell activity when VCMX was used to deliver recombinant human platelet–derived growth factor‐BB (rhPDGF) (Agis et al. 2014). rhPDGF has been found to promote angiogenesis and fibroblast proliferation, thereby accelerating wound healing (Hom and Maisel 1992; Tavelli et al. 2020a), and has gained increasing attention in both regenerative and root coverage procedures (Barootchi et al. 2025; McGuire and Scheyer 2006; McGuire et al. 2009; Tavelli, Barootchi, Rasperini, and Giannobile 2023; Tavelli et al. 2020a).

Our group conducted a triple‐blind, randomised, controlled, clinical trial (RCT) treating MAGRs with VCMX, soaked either in saline or rhPDGF (Tavelli et al. 2022). At 6 months, the rhPDGF group showed significantly higher mean root coverage (mRC), complete root coverage (CRC) and superior aesthetic and volumetric outcomes (Tavelli et al. 2022). Nevertheless, the stability of root coverage outcomes following VCMX, with or without rhPDGF, remains unknown. Therefore, this study aimed to assess the 3‐year stability of the root coverage outcomes following soft‐tissue augmentation with VCMX + saline or VCMX + rhPDGF.

## 2 Materials and Methods

### 2.1 Study Design and Trial Registration

**FIGURE 1:**
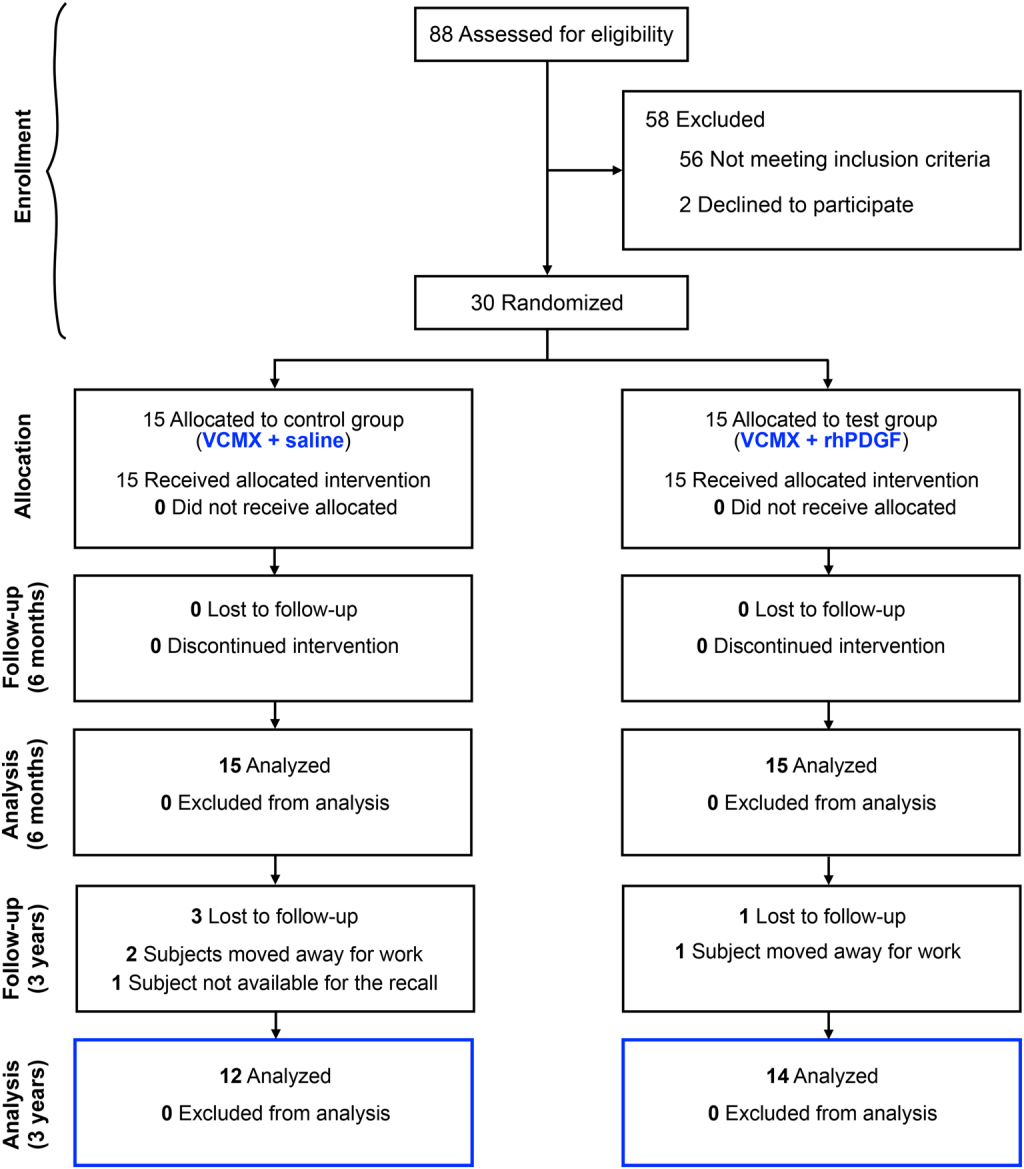
CONSORT flowchart of the study.

The present study investigates the 3‐year outcomes of a triple‐blind, parallel‐arm, randomised, placebo‐controlled clinical trial assessing the efficacy of rhPDGF in combination with a VCMX (VCMX + rhPDGF; test group) versus VCMX alone (VCMX; control group) for the treatment of MAGRs. The 6‐month outcomes had been previously reported (Tavelli et al. 2022). The clinical trial was registered prior to initiation at ClinicalTrials.gov (NCT04462237) and follows the CONSORT statement (Schulz et al. 2010) (Figure 1). The protocol of this 3‐year study was approved by the Institutional Review Board of the University of Michigan Medical School (HUM00146261), in accordance with the Declaration of Helsinki of 1975, revised in Fortaleza in 2013. Informed consent was obtained from all participating individuals in this research.

### 2.2 Participants and Intervention

**FIGURE 2:**
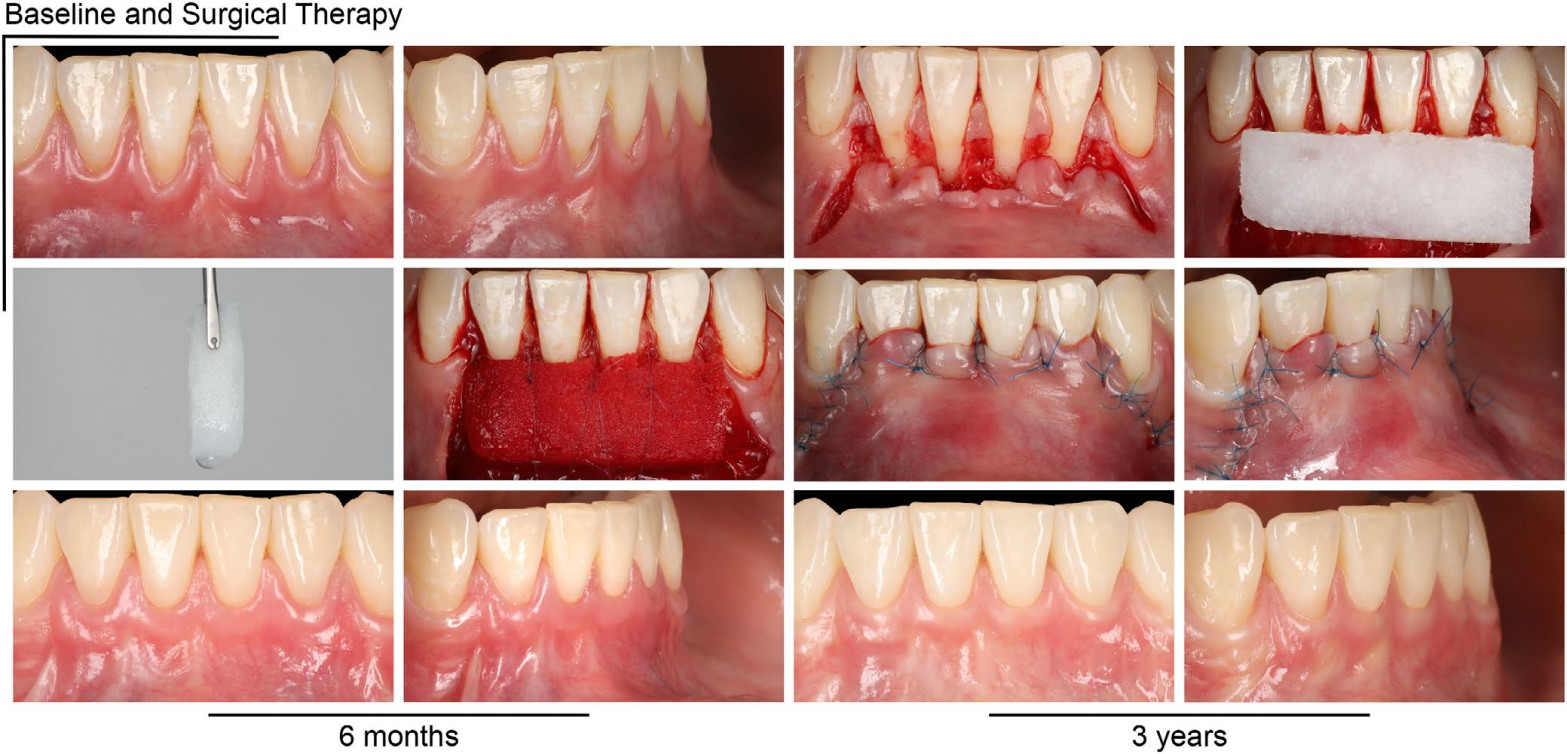
Surgical intervention at multiple mandibular gingival recessions.

Details on the inclusion and exclusion criteria are described in the original publication (Tavelli et al. 2022) and in the Appendix S1. The root coverage procedures were performed using a coronally advanced flap (CAF) in combination with a VCMX (Geistlich Fibro‐Gide, Geistlich Pharma AG, Wolhusen, Switzerland), either loaded with a sterile saline solution (vehicle control group) or with rhPDGF‐BB (GEM 21S, Lynch Biologics, Franklin, TN, USA, test group). The graft was trimmed extraorally and then saturated with a micro‐injection needle containing 1.5 mL of the solution that was prepared and provided by a masked study member. After incubating the graft in dappen dishes for 15 min, the solution was also applied onto the dried root surfaces before stabilising the graft. The flap was released and then coronally advanced to completely cover the graft and the cemento‐enamel junction (CEJ) (Figure 2 and Appendix S1).

### 2.3 Study Outcomes

The triple‐blinding protocol adopted in the original trial was preserved through the 3‐year follow‐up period, ensuring that patients, clinicians performing the procedures and outcome assessors remained blinded to group assignments. The primary endpoint of this investigation was the mRC outcomes of both control and test groups at 3 years, relative to the 6‐month results. mRC conveys the percentage of defect coverage compared to baseline.

The secondary outcomes included assessment of changes (from 6 months to 3 years) for the following parameters: (i) frequency of CRC; (ii) recession depth (Rec depth); (iii) keratinised tissue width (KTW); (iv) gingival thickness (GT); (v) midfacial probing depth (PD) and clinical attachment level (CAL); (vi) soft‐tissue volume changes using an intraoral optical scanner; (vii) aesthetic outcomes, assessed using the Root Coverage Aesthetic Score (RES) (Cairo et al. 2009); and (viii) patient‐reported outcome measures (PROMs). Additional study outcomes comprised the number of sites with stable gingival margin (GM) from 6 months to the 3‐year follow‐up, ultrasonographic mean pixel/echo intensity (EI), ultrasonographic tissue perfusion (in terms of colour Doppler velocity [CDV] and power Doppler imaging [PDI]) and strain elasticity (in terms of strain ratio [SR] based on the elasticity of the coronal soft tissue [cST] and the clinical crown [Cr]). Additional information is provided in the Appendix S1. These outcomes were assessed at the midfacial aspect of all the treated sites, as well as at their respective contralateral untreated teeth.

PROMs involved (i) postoperative morbidity, assessed using a questionnaire with a 100‐mm visual analogue scale (VAS) completed daily for 15 postoperative days (Moradi et al. 2025); (ii) patient‐reported aesthetics (EST), evaluated using a 0–100 VAS; (iii) dentin hypersensitivity (DH), assessed using the air spray approach (Meza‐Mauricio et al. 2021) and a 100‐mm VAS for each site; and (iv) overall treatment satisfaction (SAT), measured on a 0–100 VAS.

### 2.4 Statistical Methods and Outcome Assessments

Sample size calculation and stratified sequential randomisation have been reported in the original publication (Tavelli et al. 2022) and the Appendix S1. Data were entered into a prefabricated spreadsheet based on patient ID and group allocation (1 or 2). Means and standard deviations (SDs) were calculated for all continuous variables. Complete root coverage (CRC) was expressed as a binary outcome, representing the percentage of sites achieving full coverage. The unit of analysis was the treated site, as each patient contributed multiple sites to the study. To appropriately account for the non‐independence of observation due to multiple sites per patient and repeated measurements over time, linear mixed‐effects models were used to assess differences in the continuous outcomes. For binary outcomes, mixed logistic regression models were applied, with coefficients exponentiated to yield odds ratios (ORs). All models included random intercepts to account for intra‐patient correlation and repeated measures across time points. Confidence intervals (CIs) were reported, and statistical significance was set at *p* < 0.05.

## 3 Results

### 3.1 Study Participants

Out of the 39 subjects who were initially treated and completed the 6‐month follow‐up study, 26 participants (mean age at baseline 39.4 ± 11.9 years, 16 females, 10 males) contributing a total of 80 gingival recession sites were also available for the 1‐year and 3‐year follow‐up visits (Figure 1, and Table S1). All the 26 participants had received at least two additional prophylaxes throughout the 3 years.

### 3.2 Stability of Mean Root Coverage and Other Clinical Parameters from 6 Months to 3 Years

**FIGURE 3:**
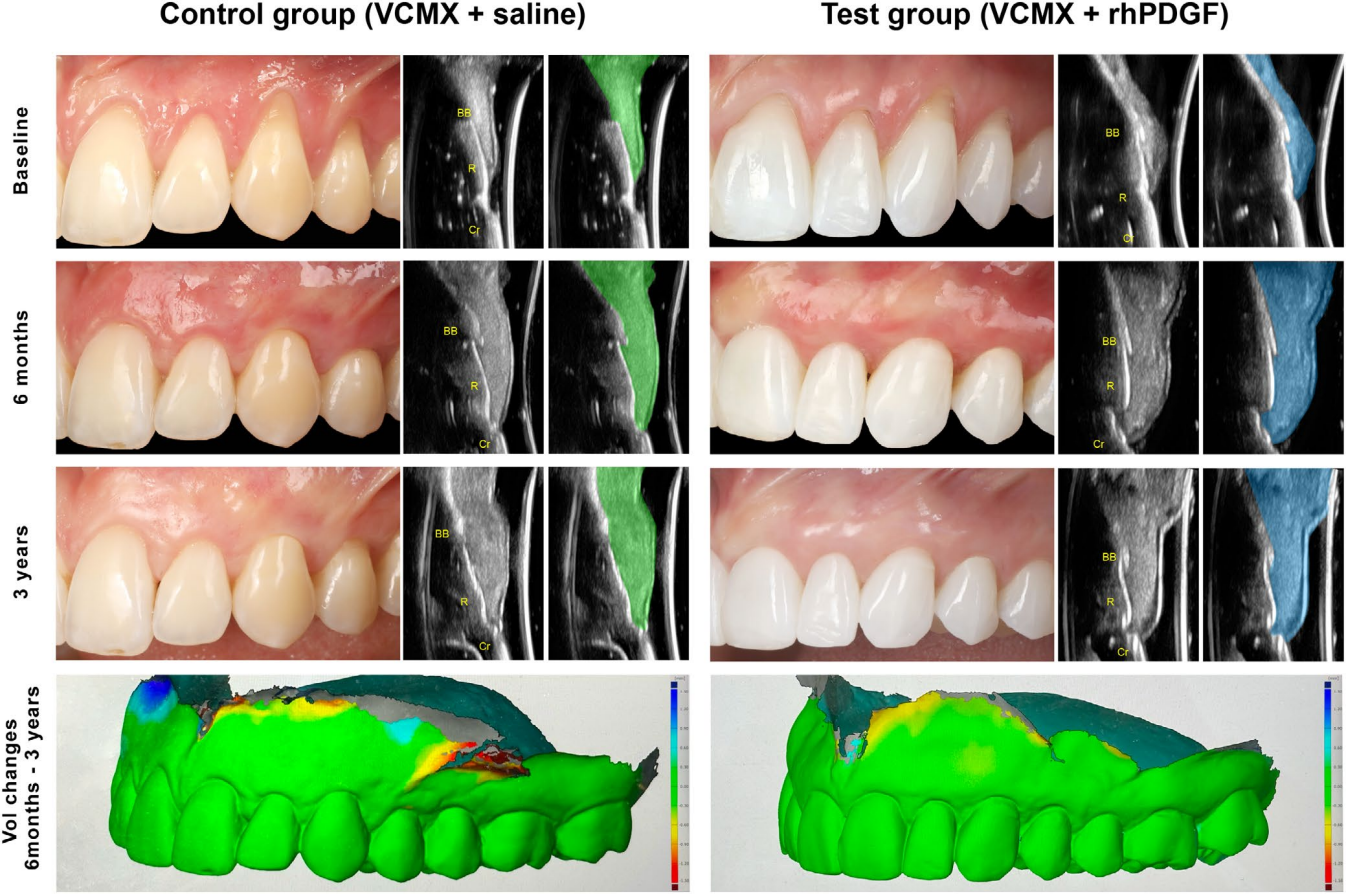
Clinical, ultrasonographic and volumetric outcomes at 6 months and 3 years following the intervention.

From 6 months to 3 years, both groups exhibited a relatively small decrease in mRC. The control group displayed a change in mRC of −6.70% from 6 months (mRC of 78.56%) to 3 years (72.67%), while the mRC change in the test group was −7.47% (mRC of 88.51% at 6 months and 81.04% at 3 years) (Tables 1 and 2). The changes in mRC were not statistically significant within the same groups over time (*p* = 0.07, −0.25 [−0.50, 0.00] for the test group and *p* = 0.10, −0.20 [−0.43, 0.04] for the control group), or between the two groups (*p* = 0.80, −0.77 [−6.67, 5.14]) (Figure 2). In both groups, more than 90% of the treated sites showed either a stable gingival margin or an apical shift of ≤ 0.5 mm from 6 months to 3 years. Both groups showed a significant increase in KTW from 6 months to 3 years (*p* = 0.03, 0.36 [0.04, 0.68] for the test group, and *p* = 0.04, 0.42 [0.03, 0.81] for the control group), while no significant changes were observed for Rec (0.53, 0.04 [−0.09, 0.17]), PD (*p* = 0.56, 0.04 [−0.08, 0.16]) or CAL (*p* = 0.36, 0.08 [−0.09, 0.24]). On the other hand, when considering the outcomes at all time points, from baseline to 3 years, regression analyses revealed that the test group outperformed the control group in terms of Rec depth reduction (*p* = 0.04, 0.20 [−0.40, −0.01]), CAL gain (*p* < 0.001, −0.46 [−0.63, −0.29]), mRC (*p* = 0.005, 7.34 [2.29, 12.38]) and CRC (p < 0.001, 3.16 [1.75, 5.69]) over 3 years (Tables 1 and 2). The mean changes in Vol observed at the test and control sites from 6 months to 3 years were −10.61 mm^3^ (*p* = 0.12, −10.61 [−22.25, 1.03]) and −9.52 mm^3^ (*p* = 0.10, −9.52 [−20.68, 1.65]), respectively. The mean changes in Δ*D* found in the test and control groups from 6 months to 3 years were −0.14 and −0.18 mm. The test group exhibited significantly greater values of Vol and Δ*D* than the control group over the 3‐year observation period (*p* = 0.04, 16.40 [0.97, 31.82] for Vol and *p* < 0.01, 0.21 [0.07, 0.34] for Δ*D*) (Tables 1 and 2, and Figure 3). The test group exhibited significantly greater scores for the level of the gingival margin (*p* < 0.001, 1.12 [0.71, 1.54]) and the final RES (*p* < 0.001, 1.22 [0.74, 1.71]) than the control group over 3 years, while the changes in RES over time within the treatment arms were not statistically significant (*p* > 0.05) (Table S2).

**TABLE 1:** Clinical and volumetric outcome measures at baseline, 6 months, 2 years and 3 years.

Outcome	VCMX + saline (12 patients, 37 sites)				VCMX + rhPDGF (14 patients, 43 sites)				*p*‐value [effect estimate (95% CI)]
	Baseline	6 months	2 years	3 years	Baseline	6 months	2 years	3 years	
Rec depth (mean ± SD) (mm)	3.16 ± 1.26	0.70 ± 0.45	0.86 ± 0.65	0.93 ± 0.66	2.88 ± 0.80	0.33 ± 0.50	0.49 ± 0.55	0.50 ± 0.55	0.04* [−0.20 (−0.40, −0.01)]
KTW (mean ± SD) (mm)	2.18 ± 1.33	2.38 ± 1.02	2.73 ± 1.02	2.80 ± 1.02	2.44 ± 0.87	2.81 ± 0.85	3.17 ± 0.85	3.17 ± 0.87	0.10 0.18 [(−0.03, 0.39)]
PD (mean ± SD) (mm)	1.42 ± 0.58	1.31 ± 0.50	1.58 ± 0.48	1.62 ± 0.57	1.38 ± 0.54	1.06 ± 0.20	1.34 ± 0.47	1.37 ± 0.54	0.65 −0.03 [(−0.16, 0.10)]
CAL (mean ± SD) (mm)	4.58 ± 1.69	1.99 ± 0.89	2.45 ± 0.84	2.55 ± 0.86	4.27 ± 0.90	1.41 ± 0.65	1.83 ± 0.70	1.91 ± 0.82	< 0.001* [−0.46 (−0.63, −0.29)]
mRC (mean ± SD) (%)	/	78.56 ± 12.26	75.15 ± 16.17	72.67 ± 17.51	/	88.51 ± 16.41	81.63 ± 19.01	81.04 ± 18.82	< 0.01* [7.34 (2.29, 12.38)]
CRC (*n*, %)	/	6, 16.22	5, 13.51	5, 13.51	/	26, 60.47	18, 41.86	18, 41.86	< 0.001* [OR 3.16 (1.75, 5.69)]
KTW gain (mean ± SD) (mm)	/	0.20 ± 1.33	0.55 ± 1.38	0.62 ± 1.37	/	0.37 ± 0.85	0.73 ± 0.90	0.73 ± 0.93	0.07* [0.22 (−0.01, 0.46)]
GT (mean ± SD) (mm)	0.91 ± 0.24	1.36 ± 0.30	1.30 ± 0.37	1.29 ± 0.42	0.95 ± 0.29	1.65 ± 0.33	1.51 ± 0.45	1.49 ± 0.47	0.02* [0.25 (0.03, 0.44)]
Vol (mean ± SD) (mm^3^)	/	58.23 ± 31.33	52.51 ± 32.56	48.72 ± 29.02	/	76.42 ± 24.47	67.85 ± 26.88	65.80 ± 24.13	0.04* [16.40 (0.97, 31.82)]
Δ*D* (mean ± SD) (mm)	/	0.70 ± 0.33	0.58 ± 0.32	0.56 ± 0.27	/	0.92 ± 0.20	0.80 ± 0.23	0.74 ± 0.23	< 0.01* [0.21 (0.07, 0.34)]

Abbreviations: CAL; clinical attachment level; CI, confidence interval; CRC, complete root coverage; KTW, keratinised tissue width; mRC, mean root coverage; *n*, number; PD, probing depth; Rec, recession; rhPDGF, recombinant human platelet‐derived growth factor‐BB; SD, standard deviation; VCMX, volume‐stable cross‐linked collagen matrix; Vol, volumetric changes assessed in mm^3^ after the superimposition of digital models; Δ*D*, mean thickness of the reconstructed volume assessed in mm after the superimposition of digital models.

* Statistically significant difference between the two groups favouring the test group.

**TABLE 2:** Changes in the clinical, volumetric and aesthetic parameters from 6 months to 3 years.

Outcome changes from 6 months to 3 years	VCMX + saline (12 patients, 37 sites)	VCMX + rhPDGF (14 patients, 43 sites)	*p*‐value [effect estimate (95% CI)]
Rec depth (mean ± SD) (mm)	0.23 ± 0.42	0.17 ± 0.33	0.49 [−0.05 (−0.19, 0.09)]
mRC (mean ± SD) (%)	−6.70 ± 12.75	−7.47 ± 13.62	0.80 [−0.77 (−6.67, 5.14)]
Sites with stable GM (*n*, %)	25, 67.57	31, 72.09	0.85
Sites with GM either stable or with an apical shift of ≤ 0.5 mm (*n*, %)	34, 91.9	41, 95.3	0.86
Sites with GM exhibiting an apical shift ≥ 1 mm (*n*, %)	3, 8.2	2, 4.7	0.86
KTW (mean ± SD) (mm)	0.42 ± 0.57	0.36 ± 0.53	0.82 [−0.02 (−0.20, 0.16)]
Vol (mean ± SD) (mm^3^)	−9.52 ± 9.28	−10.61 ± 9.74	0.61 [−1.09 (−5.35, 3.16)]
Δ*D* (mean ± SD) (mm)	−0.14 ± 0.13	−0.18 ± 0.13	0.15 [−0.04 (−0.10, 0.02)]

*Note*: Chi‐square test was used for comparing test and control in terms of sites with stable GM, sites with GM either stable or with an apical shift of ≤ 0.5 mm and sites with GM exhibiting an apical shift ≥ 1 mm.

Abbreviations: CI, confidence interval; GM, gingival margin; KTW, keratinised tissue width; mRC, mean root coverage; *n*, number; Rec, recession; rhPDGF, recombinant human platelet‐derived growth factor‐BB; SD, standard deviation; VCMX, volume‐stable cross‐linked collagen matrix; Vol, volumetric changes assessed in mm^3^ after the superimposition of digital models; Δ*D*: mean thickness of the reconstructed volume assessed in mm after the superimposition of digital models.

### 3.3 Ultrasonographic Outcomes

**FIGURE 4:**
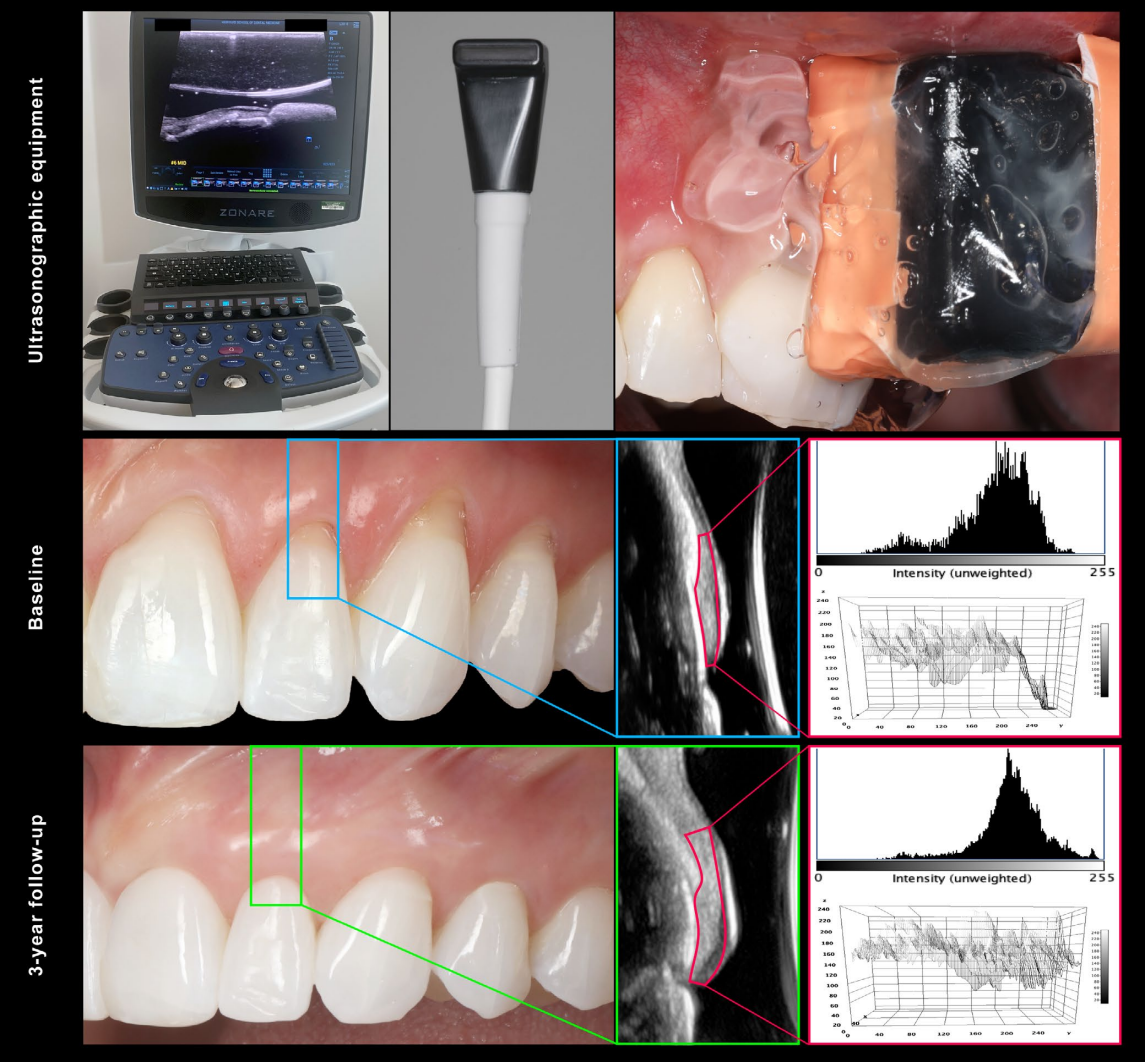
Ultrasonographic grey‐scale analysis at the regions of interest. In this example, it is possible to appreciate that the mean echo intensity (EI) of the coronal soft tissue of the lateral incisor prior to the intervention was 127.2 GL, while at the 3‐year follow‐up the respective EI was 164.4 GL, indicating a substantial change within the structure and density of the augmented soft tissue. Adapted with permission from John Wiley and Sons (Mascardo et al. 2024).

**FIGURE 5:**
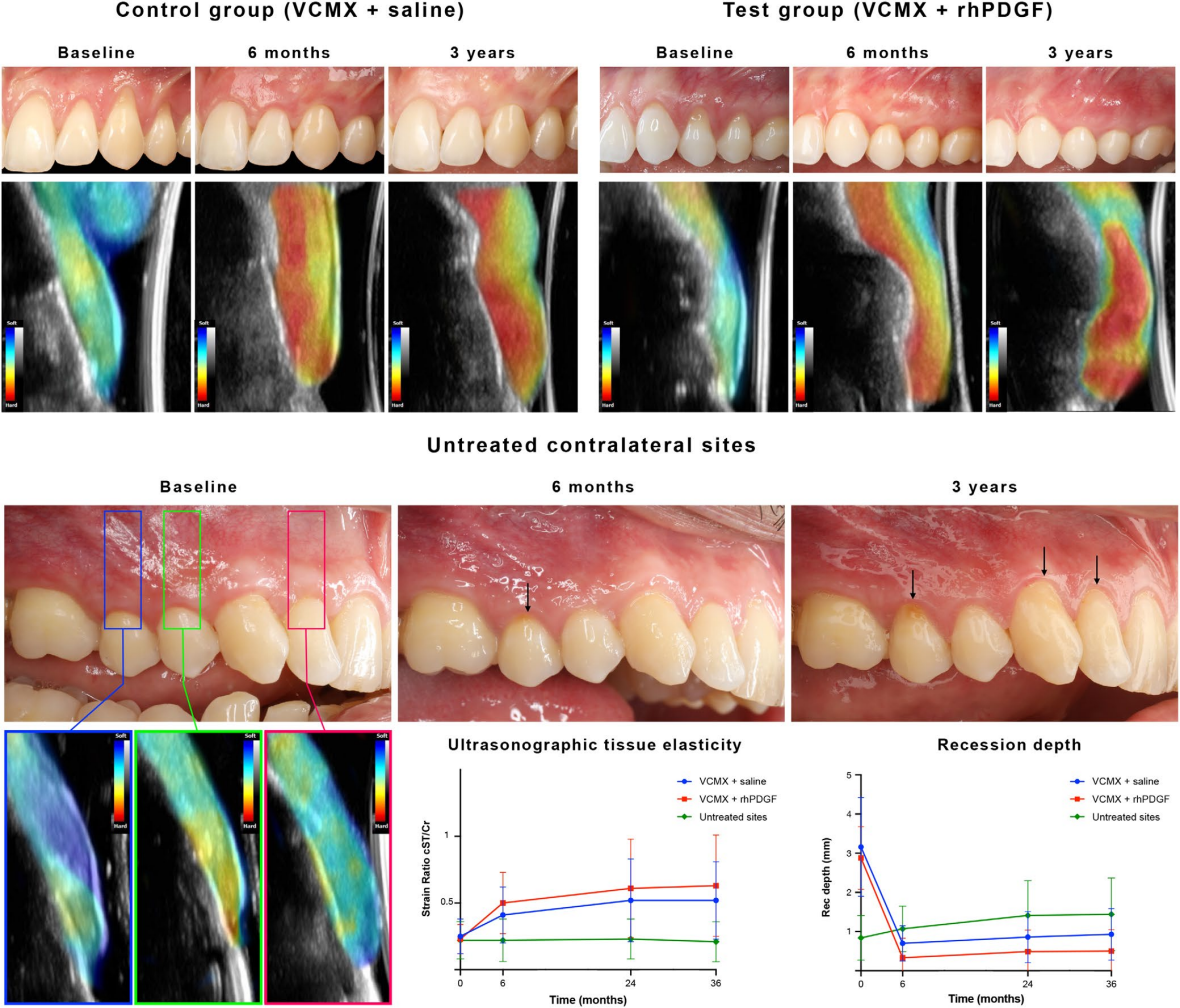
Ultrasonographic tissue elasticity changes over 3 years at the treated teeth and at their untreated contralateral sites. A significant increase in tissue elasticity was observed in both interventional groups, suggesting that root coverage procedures with a VCMX, either with or without rhPDGF, were able to promote soft‐tissue phenotypical changes within the treated sites. These changes were characterised by increased stiffness and resistance to compression compared to baseline. In contrast, untreated contralateral sites did not show significant changes in tissue elasticity but showed a significant increase in recession depth over time. In the example shown in the lower panel, sites that experienced an apical shift of the gingival margin over the 3‐year period were characterised by relatively low tissue stiffness in the coronal portion of the gingiva, indicating a ‘softer’ rather than ‘stiffer’ soft tissue.

Ultrasonographic outcomes are reported in detail in Tables S3–S5 and illustrated in Figures 3, 4, 5. Except for measurements at baseline, the test group exhibited a significantly greater GT than the control group at all investigated time points (*p* = 0.02, 0.25 [0.03, 0.44]). The mean GT reduction from 6 months to 36 months in the test and control groups was 0.16 and 0.07 mm, respectively (*p* > 0.05). Similarly, no significant changes were observed in the two groups in terms of baseline bone dehiscence (BBD) from baseline to 3 years (*p* = 0.79, −0.33 [−2.77, 2.11]). Both groups showed a significant increase in EI values over 3 years (*p* < 0.01, 36.33 [15.40, 57.27]), with no significant differences between VCMX + saline and VCMX + rhPDGF (*p* = 0.99, −0.13 [−18.71, 18.45]) (Figure 4). The regression analysis did not reveal significant changes between the two groups over 3 years for the tissue perfusion outcomes (*p* > 0.05, Figure S1).

The two groups exhibited a significant increase of ultrasonographic strain ratio cST/Cr over 3 years, indicating that the coronal soft tissue became stiffer with the surgical intervention (*p* < 0.001, 0.011 [0.009, 0.014] for the VCMX + rhPDGF group, and *p* < 0.001, 0.011 [0.008, 0.014] for the VCMX + saline group). The rate of improvement of strain ratio cST/Cr over 3 years was not statistically significantly different between the test and control groups (*p* = 0.81, 0.00 [−0.003, 0.004]) (Figure 5 and Table S5).

### 3.4 PROMs

No significant changes were found between the test and control groups from 6 months to 3 years in terms of SAT (*p* = 0.65, 0.78 [−2.55, 4.12]). However, when evaluating the entire 3‐year period, SAT scores were significantly higher in the test group (*p* < 0.001, 6.30 [2.96, 9.65]). No significant differences were detected between the two arms in terms of EST when considering all time points (*p* = 0.22, −2.00 [−5.21, 1.20]). Both groups showed a significant and sustained reduction in DH over 3 years (*p* < 0.001, −22.43 [−28.08, 16.78] for the test group and *p* < 0.001, −23.56 [−30.22, −16.89] for the control group), with no notable differences between them. Post‐surgical morbidity ratings and willingness to repeat the procedure were similarly high across groups (*p* > 0.05), with over 90% of patients in each group willing to undergo the treatment again and most reporting they would recommend it to others (*p* > 0.05) (Appendix S1 and Table S6).

### 3.5 Outcome Measures and Changes Over Time at Untreated Contralateral Sites

Untreated contralateral teeth showed a progressive and statistically significant increase in recession depth over 3 years (*p* < 0.001, 0.65 [0.42, 0.88]). No notable changes in KTW or GT were detected. EI slightly declined at untreated sites from 6 months to 3 years (*p* = 0.035, −19.34 [−37.30, −1.39]), in contrast to the increase observed at treated sites. BBD was significantly lower at untreated sites, but the change in BBD over time did not differ significantly between groups (Appendix S1 and Table S4).

Tissue elasticity analysis revealed that strain ratio values were significantly lower at untreated sites compared to both treatment groups (*p* < 0.001, 0.157 [0.103, 0.211] compared to the test group, and *p* = 0.006, 0.078 [0.022, 0.134] compared to the control group), which showed substantial improvement over time (*p* < 0.001). DH increased notably at untreated sites over 3 years, whereas it decreased in treated ones, with statistically significant differences (*p* < 0.001, −39.06 [−46.88, −31.24] for test vs. untreated sites, and *p* < 0.001, −40.19 [−48.40, −31.97] for control vs. untreated sites). Similarly, patient‐reported EST at 3 years was significantly higher for treated sites compared to their contralateral counterparts (*p* < 0.001, 73.78 [61.87, 85.69] for test vs. untreated sites, and *p* < 0.001, −40.19 [53.06, 78.10] for control vs. untreated sites) (Appendix S1 and Tables S5–S7).

## 4 Discussion

This study evaluated the medium‐term (6 months to 3 years) stability of root coverage outcomes in sites treated with VCMX, either soaked in saline or rhPDGF. Across all sites, regardless of group allocation, minimal and non‐statistically significant changes were observed in the position of the gingival margin over time. mRC decreased by 6.7% in the control group and 7.5% in the test group, without significant intra‐ or inter‐group differences. Although the test group exhibited significantly higher mRC values at 3 years compared to the control (81.0% vs. 72.7%), the changes from 6 months to 3 years were not significantly different between the groups. Most treated sites showed no changes or an apical shift ≤ 0.5 mm in the gingival margin, indicating that the soft‐tissue position remained largely unchanged during this period.

The behaviour of the gingival margin following root coverage has been widely discussed in recent literature (Barootchi et al. 2019; Pini Prato, Franceschi, et al. 2018; Pini Prato, Magnani, and Chambrone 2018; Rasperini et al. 2018). Sites treated with CAF alone tend to show apical shift over time, especially in the presence of risk factors such as narrow KTW, limited GT and interproximal attachment loss (Barootchi et al. 2019; Pini Prato, Magnani, and Chambrone 2018; Tavelli, Barootchi, Cairo, et al. 2019). Conversely, procedures involving CTG have demonstrated better long‐term stability of the gingival margin, with minimal relapse (Barootchi et al. 2019; Cairo et al. 2023; Pini Prato, Franceschi, et al. 2018; Rasperini et al. 2018; Tavelli, Barootchi, Cairo, et al. 2019). A 10‐year study reported improved long‐term stability of the gingival margin and CRC when CAF was combined with CTG (Cairo et al. 2023). In contrast, acellular dermal matrices have shown variable long‐term root coverage outcomes (Barootchi, Tavelli, Di Gianfilippo, et al. 2021; Barootchi, Tavelli, Gianfilippo, et al. 2021; Moslemi et al. 2011; Tavelli, Barootchi, Di Gianfilippo, et al. 2019), highlighting the need for further investigation, especially regarding newer materials like second‐generation xenogeneic collagen matrices (VCMX).

Previous studies involving the first‐generation collagen matrix (CMX) reported stable outcomes at 3 years (Jepsen et al. 2017; McGuire and Scheyer 2016; Tonetti et al. 2021), while others demonstrated moderate mRC decreases over time (Molnar et al. 2022). A 3‐year multi‐centre trial showed that mRC dropped from 69.2% to 57.7% (Tonetti et al. 2021), while another study found an 11.9% decrease over 5 years (McGuire and Scheyer 2016). A 9‐year study revealed even more significant mRC decrease (approximately 50%) using the tunnel technique and CMX. Until now, medium‐ and long‐term root coverage outcomes for the newer VCMX were limited to 6‐ and 12‐month evaluations. To our knowledge, this is the first randomised clinical trial reporting 3‐year outcomes for CAF + VCMX.

The gingival margin stability in this study is likely due to soft‐tissue phenotype modification promoted by the graft (Barootchi et al. 2020). In previous work, we demonstrated that sites with at least 1.5 mm of KTW and ≥ 1.47 mm GT at 6 months had the highest probability of maintaining the gingival margin stable over 10 years. In this cohort, VCMX yielded average GT gains of 0.45 mm (control) and 0.7 mm (test) after 6 months, supporting its role in phenotype enhancement. These gains, combined with an existing adequate band of KTW, likely led to a thicker and stable gingival margin. Supporting these findings, studies by Agudio et al. demonstrated that gingival phenotype modification ensured gingival margin stability for up to 30 years (Agudio et al. 2017; Agudio et al. 2019). While VCMX + rhPDGF led to superior outcomes at 6 months, 2 years and 3 years—such as higher mRC, recession reduction, CRC and aesthetic scores—these differences likely stemmed from the initial influence of rhPDGF on early healing. This aligns with the known biological actions of rhPDGF, which include promoting angiogenesis, accelerating inflammation resolution and stimulating fibroblast activity and matrix production (Cheng et al. 2007; Cooke et al. 2006). The similarity in clinical performance between the two groups from 6 months to 3 years suggests that rhPDGF's primary effects occur during the early healing phase.

Several approaches have explored the use of collagen matrices with biologics to enhance root coverage outcomes (Cardaropoli et al. 2025; Krishnaraj et al. 2023; Michels et al. 2023; Rossato et al. 2024; Sangiorgio et al. 2017; Santamaria et al. 2023). Sangiorgio et al. reported favourable outcomes with CAF + CMX + enamel matrix derivative (EMD), although similar results were achieved using CMX or EMD alone (Sangiorgio et al. 2017). More recently, injectable platelet‐rich fibrin (i‐PRF) has been applied to collagen scaffolds, with case series reporting mRCs between 71.7% and 81.1% and CRC around 40% (Michels et al. 2023; Rossato et al. 2024). A combination of VCMX with polynucleotides and hyaluronic acid yielded 12‐month outcomes of mRC 96% and CRC 80.6% (Cardaropoli et al. 2025). While direct comparisons are limited by study design and follow‐up duration, these findings support VCMX as a promising scaffold for biologic delivery. This is consistent with the American Academy of Periodontology Best Evidence Consensus report, which endorses the use of biologics and soft‐tissue graft substitutes to enhance healing and root coverage outcomes, particularly when CTG is not used, or in complex cases (Avila‐Ortiz et al. 2022).

Importantly, untreated contralateral sites showed an average gingival recession increase of 0.6 mm over 3 years. This is consistent with Rios et al., who reported a 0.41 mm increase over 4 years at untreated teeth (Rios et al. 2021). In our cohort, several factors may have contributed to this apical shift, such as the high prevalence of thin and medium phenotypes, limited GT and bone dehiscence (Mascardo et al. 2024; Romandini et al. 2020). Although the treated sites exhibited similar baseline characteristics, the augmentation with a VCMX, either with saline or rhPDGF, was able to significantly improve their root coverage and soft‐tissue phenotype. Ultrasonographic analysis further revealed structural and mechanical differences between treated and untreated sites. EI, which reflects tissue density (Galarraga‐Vinueza et al. 2024; Mancini et al. 2023; Tavelli, Majzoub, Kauffmann, et al. 2023), progressively increased in VCMX‐treated sites, while untreated sites showed slight EI reductions. Although histological validation is needed, this suggests a densification of the soft tissue post treatment. Tissue elasticity assessment confirmed these changes: strain ratio progressively increased in both treatment groups, indicating stiffer, more mature soft tissue in the coronal region.

Additionally, while not statistically significant, sites treated with VCMX + rhPDGF showed better PROMs at 3 years, specifically higher EST and reduced DH. Meanwhile, untreated contralateral sites showed significant worsening in both parameters. These differences further support the clinical relevance of root coverage procedures and soft‐tissue phenotype modification.

This study has some limitations. Four of the original 30 patients were lost to follow‐up at 2 and 3 years. These missing data could have impacted the results. Also, although rhPDGF showed clinical advantages, this study did not include a formal cost versus effectiveness analysis. Future studies incorporating economic evaluation models may help define the clinical and financial justification for the use of rhPDGF and other biologics in root coverage procedures.

## 5 Conclusion

This 3‐year RCT demonstrated that the gingival margin of sites treated with either VCMX + saline or VCMX + rhPDGF remained largely stable between 6 months and 3 years. Minor, non‐significant changes were observed in clinical, volumetric and ultrasonographic parameters, with no notable differences between the two treatment groups. Augmented sites exhibited a progressive increase in ultrasonographic EI and tissue elasticity, indicating that the coronal soft tissue became denser and stiffer as a result of soft‐tissue phenotype modification. Untreated contralateral teeth showed a tendency towards the apical shift of the gingival margin over 3 years, together with an increase in patient‐reported DH.

## Author Contributions

L.T.: conceptualisation, recruitment, surgical procedures, manuscript writing, final approval. S.B.: conceptualisation, randomisation, data analysis, manuscript writing, final approval. M.V.R.: recruitment, patient visits, manuscript revision, final approval. L.M.: 3D analysis, manuscript writing, final approval. H.S.: patient visits, final approval. T.N.: ultrasound blood flow analysis, manuscript revision, final approval. J.M.: Recruitment, data collection, ultrasound analysis, manuscript revision, final approval. S.T.: Recruitment, patient visits, manuscript revision, final approval. W.V.G.: Conceptualization, manuscript writing, critical review and final approval.

## Conflicts of Interest

L.T. and S.B. have previously received honoraria and grants from Geistlich Pharma. The other authors do not have any financial interests, either directly or indirectly, in the products or information listed in the paper.

## Supporting information


**Appendix S1:** Supporting Information.

## Data Availability

The data that support the findings of this study are available from the corresponding author upon reasonable request.
