# Supplementary material for: Stability of Root Coverage Outcomes After Soft‐Tissue Augmentation With a Collagen Matrix With or Without rhPDGF‐BB: A 3‐Year Triple‐Blinded, Randomised, Placebo‐Controlled Trial
Source: J Clin Periodontol. 2025 Sep 11;52(12):1734–45. doi: 10.1111/jcpe.70030 (PMC12605705; doi:10.1111/jcpe.70030)
Supplement: Supplementary file 1 — Appendix S1: Supporting Information. [file JCPE-52-1734-s001.docx]

**Stability of root coverage outcomes after soft tissue augmentation with a collagen matrix with or without rhPDGF-BB: A 3-year triple-blinded, randomized, placebo-controlled, trial**

Lorenzo Tavelli, Shayan Barootchi, Maria Vera Rodriguez, Leonardo Mancini, Hamoun Sabri, Tu Nguyen, Jad Majzoub, Suncica Travan, and William V. Giannobile

**Supplementary Appendix**

**Supplementary Material and Methods**

**Patient recruitment and inclusion/exclusion criteria**

Participants were recruited based on the following inclusion criteria: i) Periodontally and systemically healthy adults (age ≥ 18 years) presenting with at least 2 MAGRs classified as recession type 1 (RT1)(Cairo, Nieri, Cincinelli, Mervelt, & Pagliaro, 2011), associated with dental hypersensitivity or esthetic concerns; ii) self-reported smoking ≤ 10 cigarettes/day; iii) full-mouth plaque and bleeding scores ≤ 20%; iv) presence of a least 2 mm depth on at least one recession, and v) patients being able to maintain good oral hygiene.

The exclusion criteria included: i) compromised general health, ii) pregnancy or attempting to get pregnant (self-reported), iii) untreated periodontal disease, iv) persistence of uncorrected factitious gingival trauma from toothbrushing, v) presence of severe tooth malposition, rotation or super-eruption, vi) presence of root caries or inadequate prosthetic restorations, vii) previous periodontal plastic surgery at the experimental sites, viii) known allergy to collagen-based medical products.

**Interventions**

Presurgical procedures were performed at least 1 month before the surgery and involved a session of dental prophylaxis with oral hygiene instructions that aimed at eliminating possible traumatic toothbrushing habits. The intervention consisted of coronally advanced flap (CAF) with a VCMX (Geistlich Fibro-Gide, Geistlich Pharma AG, Wolhusen, Switzerland), either saturated with a sterile saline solution (vehicle control group) or with rhPDGF-BB (GEM 21S, Lynch Biologics, Franklin, TN, USA, test group). Based on the location and distribution of the MAGRs, CAF was performed with a trapezoidal or envelope design, with horizontal or rotated papillae, with or without vertical incisions, as previously described (Tonetti et al., 2018; Zucchelli & De Sanctis, 2000; Zucchelli et al., 2009). Mechanical and chemical root conditioning was performed with mini curettes and 24% of EDTA for 2 minutes, respectively. The VCMX was trimmed extraorally with a 15c blades, based on the size characteristics of the recipient bed, and then it was saturated with a micro-injection needle containing 1.5 cc of the solution that was prepared and provided by masked study member through a sealed envelope. The matrices were incubated in dappen dishes for 15 minutes prior to delivery to the surgical site (Rubins, Tolmie, Corsig, Kerr, & Kim, 2013, 2014). The solution was also applied onto the dried root surfaces before stabilizing the graft. Simple interrupted sutures (6/0 and 7/0 PGA, AD Surgical, Sunnyvale, USA) engaging the VCMX and the de-epithelialized anatomical papillae were performed for stabilizing the graft at the level of the cemento-enamel junction (CEJ) or 1 mm apical. Further stabilization of the matrix was also achieved, if necessary, with additional mattress sutures apical to the VCMX, through engaging the periosteum. The flap, that was previously released, was then coronally advanced and stabilized approximately 2 mm above the CEJ using sling sutures and simple interrupted sutures (6/0 and/or 7/0 polypropylene [Ethicon, Johnson & Johnson, Somerville, USA]) at the level of the papillae. Simple interrupted sutures were performed at the level of the vertical incisions, if any (7/0 polypropylene [Ethicon, Johnson & Johnson, Somerville, USA]) (Tavelli et al., 2022) (Fig. 2).

**Clinical measures**

The same masked and calibrated examiner who performed the clinical assessments in the original study also conducted the evaluations at the 2- and 3-year follow-up visits, and was re-calibrated prior to each time point. The calibration consisted of two repeated measurements of REC and KTW among 10 subjects who had not participated in the study (K coefficient of 0.90 for REC and of 0.87 for KTW, for obtaining measurements within 0.5 mm). The following clinical measurements were assessed and recorded at baseline, 6 months, 2 years and 3 years after the surgery on the mid-buccal aspect of all treated sites, using a periodontal probe (PCP UNC 15, Hu-Friedy, Chicago, IL, USA) as previously described (Cairo et al., 2016; Tavelli et al., 2022): i) Recession depth (Rec), ii) Probing depth (PD), iii) Clinical attachment level (CAL) and iv) Keratinized tissue width (KTW). Gingival phenotype was classified at each site as thin, medium, thick, or very thick using color-coded probes (Colorvue probes, Hu-Friedy, Chicago, IL, USA). The professional esthetic assessment was performed at 6 months, 2 years and 3 years utilizing the Root coverage Esthetic score (RES) (Cairo, Rotundo, Miller, & Pini Prato, 2009).

**STL file acquisition and Volumetric outcome assessment**

Digital models were obtained using an intraoral optical scanner (Trios, 3Shape, Denmark) at baseline, 3 months, 6 months, 2 years and 3 years. The models were saved as STL files and imported in an image analysis software (GOM Inspect, GOM, Germany) at the end of the study. A blinded and pre-calibrated examiner with experience in 3D volumetric analysis (L.M.) performed all the measurements. A semi-automated alignment, based on the selection of reproducible points on the digital models and on a best-fit algorithm, was used to superimpose the STL files (Borges et al., 2020; Parvini et al., 2021). Each time point was superimposed with the baseline, which was used as the reference. The region of interest (ROI) was defined as previously described at each treated site (Tavelli, Barootchi, Majzoub, Siqueira, et al., 2021). The volumetric outcomes of interest were volume change in mm^3^ (Vol) and the mean thickness of the reconstructed volume in mm (ΔD) (Fons-Badal et al., 2020; Schmitt et al., 2016; Tavelli, Barootchi, Majzoub, Siqueira, et al., 2021; Tian et al., 2019; Xue et al., 2021) (Fig. 3).

**Ultrasound image acquisition**

A commercially available ultrasound imaging device (ZS3, Mindray) was coupled with a 24 MHz (64 μm axial image resolution) and miniature-sized (approximately 30 mm long, x 18 mm wide x 12 mm thick) probe (L30-8) to generate ultrasound images. Single image frames (“still images”) at the mid-facial aspect of the site of interest were saved in “B-mode” in the Digital Imaging and Communications in Medicine (DICOM) format. “B-mode” generates 2D grey-scale images in which brightness is the result of the returned echo signal and its strength, which depends on the acoustical properties of the periodontal soft and hard tissues. The US probe was oriented perpendicular to the occlusal plane and parallel to the long axis of the tooth at its midfacial aspect (Chan & Kripfgans, 2020b; Tavelli, Barootchi, et al., 2021b) (Fig. 3-5, and Supplementary Fig. 1).

The ultrasonographic analyses were performed by the same masked and precalibrated operator (J.M.) who assessed the ultrasonographic outcomes in the original study. Prior to analyzing the 2- and 3-year follow-up data, the operator was re-calibrated by repeating a set of measurements (GT, BBD, pRI, and Strain ratio cST/Cr) on 10 isolated gingival recessions from 10 subjects that were not included in the present study. All assessments were conducted twice, two weeks apart. The intraclass correlation coefficients (ICC) for these outcomes were ≥ 0.85.

**Ultrasonographic linear and gray-scale outcomes**

The ultrasound equipment setup and the scanning procedures have been described in detail in previous reports (Chan & Kripfgans, 2020a; Mascardo et al., 2024). Ultrasound scans were obtained at baseline, 2 weeks, 3 months, 6 months, 2 years, and 3 years. The DICOM files obtained from the scans were imported in a public-domain software package (Horos^TM^, version 4.0.0, Horos Project) to evaluate gingival thickness (GT) 1.5 from the gingival margin, and buccal bone dehiscence/distance (BBD), measured as the distance between the bone crest and the CEJ (Chan et al., 2017; Tattan et al., 2019) (Fig. 3). The scans were also exported in JPEG format and then imported into another public-domain software package (ImageJ, National Institutes of Health, Bethesda, MD, USA), where the gray texture analysis. The region of interest (ROI) was defined on the soft tissue as the area extending from the gingival margin for 7 mm in a corono-apical direction (Galarraga-Vinueza et al., 2024). The mean pixel/echo intensity (EI) was computed from the histograms generated by the software for the selected ROI (Galarraga-Vinueza et al., 2024; Kim, Kim, Moon, Yoon, & Kwak, 2015; Mancini, Khehra, Nguyen, Barootchi, & Tavelli, 2023) (Fig. 4).

**Ultrasonographic tissue perfusion assessment**

Tissue perfusion was assessed and recorded as six second cine loops of Color Doppler Velocity (CDV) and Power Doppler Imaging (PDI) modalities capturing at least 5 cardiac cycles (Barootchi et al., 2022; Tavelli, Barootchi, et al., 2021a; Tavelli et al., 2023). CDV mode allows to detect mean velocity of blood flow within vessel through color coding, based on the scattered signal produced by moving erythrocytes that results in a change in frequency of the reflected sounds waves that are received by the ultrasound transducer. This imaging modality provides information on blood flow direction and relative velocity, while PDI displays in a single-hue red color the power/amplitude of the blood flow.

The DICOM files containing CDV cine loops recorded at the midfacial aspect of each site of interest at baseline, 3 months, 6 months, 2 years, and 3 years were imported in a software package (PixelFlux, version 2018, Chameleon-Software, Germany) where a masked and precalibrated operator with expertise in dental ultrasonography (T.N.) performed the dynamic tissue perfusion measurements (DTPMs) of interest (Dyrla et al., 2019; Rosenbaum et al., 2013; Scholbach, Girelli, & Scholbach, 2005; Scholbach & Scholbach, 2009). The calibration consisted in evaluating DTPMs on 10 grafted sites from 10 subjects that were not included in the present study. All assessments were conducted twice, two weeks apart. The intraclass correlation coefficients (ICC) for these outcomes were ≥ 0.82.

The region of interest (ROI) for the DTPMs was determined within the soft tissue, extending from the gingival margin to a point 7 mm apical to it (Tavelli, Barootchi, et al., 2021a; Tavelli et al., 2023). B-mode scans were utilized to identify the boundaries of the soft tissue. The following DTMs of interested were automatically computed by the software for both ROIs (Supplementary Fig. 1):

- Mean perfusion relief intensity (pRI), which was obtained from the “perfusion relief” function of the software, which provides a visual impression of the vasculature depicting the local distribution and intensity of the perfusion within the ROI (Rosenbaum et al., 2013; Scholbach et al., 2005; Scholbach & Scholbach, 2009; Tavelli et al., 2023)
- Flow intensity (FI), which was calculated by the software with the formula:

$$FI \left( cm/s \right)=\frac{velocity \left( cm/s \right) x A ({cm}^{2})}{A_{ROI} ({cm}^{2})}$$

where velocity corresponds to the color hue of the pixels within the selected ROI, “A” is the mean perfused area determined by the number of perfused pixel within the ROI, and A_ROI_ is the total area of the ROI. FI increases with the perfusion velocity, and decreases if the perfused area is less than the area of the ROI. FI is intended to increase the objectivity and comparability of perfusion data, as it corresponds to the average velocity, provided that the whole region of interest is perfused homogenously. FI was obtained as FI_red_, capturing the FI of the blood flowing towards the transducer only, FI_blue_, capturing the perfusion intensity of the blood flowing away from the transducer only, and FI_mix_, calculated as the average between FI_red_ and FI_blue_. These outcomes were calculated both CDV and PDI cine loops. For the PDI cine loops, FI could only be expressed as FI_red_.

**Tissue elasticity analysis**

The methodology for assessing strain tissue elasticity has been depicted in detail in a recent publication (Tavelli & Barootchi, 2024). Briefly, the AVI files containing the cine loops depicting the compression of the tissue were imported in an advance intelligence elastography software (US-Elasto, Dileny Technologies and biomedical engineering LCC, Giza, Egypt) that adopts a patented method to reconstruct strain elastography from gray-scale imaging videos (Kamal, Kamal, Mahmoud, Mekhaimar, & Hanafy, 2022; Mahmoud & Ali, 2020) . The frame-to-frame displacement were calculated using hierarchy recursive displacement tracking technique, with the strain that was computed as displacement spatial derivative and then superimposed on B-mode scans to visualize anatomical and elastography outcomes using a color-coded map (elastogram) depicting the different grades of elasticity, from soft to hard (Fig. 5) (Kamal et al., 2022). Two standardized regions of interest (ROIs) were identified within the same cine loop video for quantifying tissue elasticity as a strain ratio (SR) (Kamal et al., 2022; Mahmoud & Ali, 2020). The most coronal portion of the soft tissue (cST) was the first ROI, while the second ROI was the crown (Cr), that was used as a fixed reference. Then, frame-to-frame strain ratio (SR) was calculated by the software with the formula:

$${SR}=\frac{{Strain}_{Cr}(\%)}{{Strain}_{cST} (\%)}$$

SR < 1 indicates that the ROI displayed as denominator in the above formula (cST) has a higher strain/elasticity and a lower stiffness (rigidity) relative to the other ROI (Cr). Values approaching 0 indicate greater elasticity within the coronal soft tissue, while values approaching 1 reflect greater stiffness in this region (Choi, Lee, & Baek, 2015; Gennisson, Deffieux, Fink, & Tanter, 2013; Sigrist, Liau, Kaffas, Chammas, & Willmann, 2017; Tavelli & Barootchi, 2024).

**Sample size**

The original 6-month study was powered to detect a minimum clinically significant difference in root coverage (recession reduction) of 0.5 mm using α = 0.05, a power (1- β) of 80%, and a hypothesized within-group sigma of 0.4 mm (Cairo et al., 2016). Considering possible dropouts, the number of patients were increased by 15% for each arm. On the basis of these data, the minimum number of patients needed to be enrolled in this study was 30 in total, 15 for the test (VCMX + rhPDGF), and 15 for the control group (VCMX + saline).

**Stratified sequential randomization**

Three sets of ten patients were stratified by a computer software to obtain two equally balanced groups (of A and B) based on baseline characteristic of initial recession depth, arch, and smoking status. By the flip of a coin of the study coordinator, it would be decided which of the two groups would serve as test (VCMX + rhPDGF), and which would be control (VCMX + sterile saline solution, as placebo).

On the day of the surgery, the surgeon would receive a sealed envelope with the patient’s ID number, containing a syringe with 1.5 cc of a clear solution which could have either been sterile saline (control), or 0.3 mg/mL rhPDGF (test group). The test and control envelopes and syringes appeared identical. The patients, the surgeon, and other study team members were unaware and remained uninformed of the test/control treatment allocation. All patients received treatment as they were assigned.

**Post-operative regimen**

Oral and written post-operative instructions were given to the patients. Patients were instructed to avoid traumas and brushing at the surgical site for two weeks and to intermittently apply an ice pack for the first 24 hours. Patients were prescribed Ibuprofen 600 mg every 4-6 hours for the first 3 days, followed by its prescription as needed for pain/discomfort and Chlorhexidine mouth rinse 0.12 % twice daily for one minute for 14 days. Smokers were reminded to quit smoking during the first two weeks. The sutures were removed two weeks after the surgical procedure. Patients were instructed to resume mechanical tooth brushing using an extra-soft bristle toothbrush at the operated area. At the 1-month appointment, patients were given a soft bristle toothbrush to replace the extra-soft bristle one, and oral hygiene instructions were reinforced. Patients also returned at 3 months, 6 months, 1 year and 3 years after the surgery.

**Supplementary Results**

**Patient-reported outcomes (PROMs)**

PROMs are depicted in detail in Supplementary Table 6. No significant changes were found between the test and control groups from 6 months to 3 years in terms of SAT (p=0.65, 0.78 [-2.55, 4.12]). However, when SAT was assessed over the 3 years, significantly higher values were found at sites allocated to the test group (p<0.001, 6.30 [2.96, 9.65]). No significant changes were observed at test sites from 6 months to 3 years in terms of EST (p=0.44, 1.47 [-2.26, 5.21]), while the EST change at the control sites from 6 months to 3 years was statistically significant (p<0.01, 6.46 [1.61, 11.32]). Nevertheless, when the regression analysis considered all time points from baseline to 3 years, no significant differences were found between test and control group for EST (p=0.22, -2.00 [-5.21, 1.20]). The two groups significantly reduced DH over 3 years (p<0.001, -22.43 [-28.08, 16.78] for the test group and p<0.001, -23.56 [-30.22, -16.89] for the control group), with the results that were found stable from 6 months to 3 years in both groups (p>0.05 for both comparisons). Similarly, no significant differences were found between the two groups for DH changes over time (p=0.51, -1.92 [-7.62, 3.77]). No differences were noticed between the test and control groups when patients were asked to rate their perception of the post-surgical morbidity (p>0.05). More than 90% of the subjects in each group reported that they would be willing to undergo the same procedure again, if necessary (p>0.05), while 83.3% of subjects in the control group and 92.9% of patients in the test group stated that they would recommend the same procedure to friends and family members (p>0.05).

**Outcome measures and changes over time at untreated contralateral sites**

Untreated contralateral sites exhibited a mean Rec depth of 0.89 mm at baseline, which became 1.06 mm at 6 months, 1.44 mm at 2 years, and 1.51 mm at 3 years. The change of Rec depth from baseline to 3 years was statistically significant (p<0.001, 0.65 [0.42, 0.88]). No significant changes were observed at untreated sites over 3 years for KTW and GT (p>0.05 for both outcomes). EI changes from baseline to 6 months were not statistically significant (p=0.81, -2.23 [-20.19, 15.72]), while EI changes from 6 months to 3 years were statistically significant (p=0.035, -19.34 [-37.30, -1.39]). The baseline BBD of the untreated contralateral sites was, on average, 4.01 mm, while at the test and control sites baseline BBD was, on average, 6.26 mm, and 6.57 mm, respectively. After 3 years, the mean BBD at untreated contralateral sites was 5.08 mm, indicating a mean BBD increase of 1.07 mm. Overall, BBD at test and control sites was significantly higher than the one at untreated contralateral sites (p=0.002, 2.25 [0.82, 3.68] for the test group and p=0.001, 2.56 [1.05, 4.06]) for the control group). However, when BBD changes over 3 years were assessed, no significant differences were found among the three groups (p=0.31, -1.04 [-3.06, 0.99] for test vs untreated sites, p=0.51, -0.71 [-2.84, 1.42] for control vs untreated sites, and p=0.79, -0.33 [-2.77, 2.11] for test vs control sites). The assessment of gingival phenotype at different time points using color-coded probes is reported in the Supplementary Table 7.

The strain ratio cST/Cr at the 3-year follow-up at the untreated sites was, on average, 0.21, while the same parameter was, on average, 0.52 and 0.63 at the sites treated with VCMX + saline and VCMX + rhPDGF, respectively. The statistical analysis revealed that untreated sites had significantly lower strain ratio cST/Cr than the test group (p<0.001, 0.157 [0.103, 0.211]) and control group (p=0.006, 0.078 [0.022, 0.134]) over time. No significant changes in terms of ultrasonographic tissue elasticity were noticed at the untreated contralateral sites over 3 years (p=0.48, 0.000 [-0.000, 0.001]), while a significant improvement of the strain ratio cST/Cr was observed at the sites augmented with VCMX + rhPDGF (p<0.001, 0.011 [0.009, 0.014]) and VCMX + saline (p<0.001, 0.011 [0.008, 0.013]).

Patients reported a mean increase of 16.7 VAS over 3 years for DH at the untreated contralateral sites, which is in contrast with the two treatment groups that exhibited a significant/substantial decrease in DH over 3 years (mean DH decrease of 22.4 VAS and 23.5 VAS in the test and control group, respectively). The difference of DH values between the treated sites and untreated contralateral sites over 3 years was statistically significant (p<0.001, -39.06 [-46.88, -31.24] for test vs untreated sites, and p<0.001, -40.19 [-48.40, -31.97] for control vs untreated sites). Similarly, the patient-reported EST at 3 years was significantly greater at the treated teeth compared to their contralateral sites (p<0.001, 73.78 [61.87, 85.69] for test vs untreated sites, and p<0.001, -40.19 [53.06, 78.10] for control vs untreated sites).

**Supplementary Table 1.** Patient and site characteristics at baseline for the subjects that completed the 3-year study.

| **Parameter** | **VCMX** | **VCMX + rhPDGF** |
| --- | --- | --- |
| Patients (N) | 12 | 14 |
| Age  (mean ± SD) (years) | 43.4 ± 11.7 | 36.0 ± 11.4 |
| Females (N)/ (%) | 6/ 50 | 10/ 71.4 |
| Smokers (≤ 10 cig/day) (N) | 0 | 0 |
| Total Sites (n) | 37 | 43 |
| Sites with NCCLs (n) | 4 | 3 |
| Sites in which the CEJ was reconstructed (n) | 4 | 3 |

**Legend**. CEJ: cemento-enamel junction; Cig: cigarettes; N/n: number; NCCL: non-carious cervical lesion; rhPDGF: recombinant human platelet-derived growth factor-BB; SD: standard deviation. VCMX: volume-stable cross-linked collagen matrix; No statistically significant differences were found between the two groups for the above mentioned parameters at baseline.

**Supplementary Table 2.** Professional esthetic outcomes at 6 months and 3 years using the Root coverage Esthetic Score (RES).

| **Parameter** | **VCMX + saline** | | | **VCMX + rhPDGF** | | | **p-value**  **[effect estimate (95% CI)]** |
| --- | --- | --- | --- | --- | --- | --- | --- |
|  | 6 months | 2 years | 3 years | 6 months | 2 years | 3 years |  |
| GM (mean ± SD) (points) | 3.49 ± 1.12 | 3.43 ± 1.06 | 3.41 ± 1.04 | 4.81 ± 1.48 | 4.47 ± 1.51 | 4.33 ± 1.51 | <0.001*  [1.12 (0.71, 1.54)] |
| MTC (mean ± SD) (points) | 0.81 ± 0.40 | 0.82 ± 0.39 | 0.81 ± 0.40 | 0.88 ± 0.32 | 0.86 ± 0.35 | 0.84 ± 0.37 | 0.40  [0.50 (-0.07, 0.17)] |
| STT (mean ± SD) (points) | 0.76 ± 0.43 | 0.78 ± 0.41 | 0.78 ± 0.42 | 0.72 ± 0.45 | 0.78 ± 0.42 | 0.79 ± 0.41 | 0.83  [-0.01 (-0.15, 0.12)] |
| MGJ (mean ± SD) (points) | 0.84 ± 0.37 | 0.89 ± 0.31 | 0.92 ± 0.28 | 0.84 ± 0.37 | 0.88 ± 0.32 | 0.93 ± 0.26 | 0.92  [0.01 (-0.10, 0.11)] |
| GC (mean ± SD) (points) | 0.92 ± 0.28 | 0.88 ± 0.32 | 0.86 ± 0.35 | 0.95 ± 0.21 | 0.95 ± 0.21 | 0.95 ± 0.21 | 0.14  [0.06 (0.021, 0.14)] |
| Final RES (mean ± SD) (points) | 6.81 ± 1.20 | 6.81 ± 1.13 | 6.78 ± 1.16 | 8.21 ± 1.96 | 7.94 ± 1.78 | 7.84 ± 1.69 | <0.001*  [1.22 (0.74, 1.71)] |

**Legend.** CI: confidence interval; GC: gingival color; GM: level of the gingival margin; MGJ: alignment of the mucogingival junction; MTC: marginal tissue contour; rhPDGF: recombinant human platelet-derived growth factor-BB; SD: standard deviation; STT: soft tissue texture. VCMX: volume-stable cross-linked collagen matrix; * Statistically significantly difference between the two groups favoring the test group.

**Supplementary Table 3**. Ultrasonographic tissue perfusion analysis

| **Outcome** | **VCMX**  **(12 patients, 37 sites)** | | | | | **VCMX + rhPDGF**  **(14 patients, 43 sites)** | | | | |
| --- | --- | --- | --- | --- | --- | --- | --- | --- | --- | --- |
|  | BL | 3M | 6M | 2Y | 3Y | BL | 3M | 6M | 2Y | 3Y |
| pRI  (mean ± SD) (cm/s) | 0.17 ± 0.12 | 0.11 ± 0.13 | 0.09 ± 0.1 | 0.07 ± 0.07 | 0.06 ± 0.07 | 0.12 ± 0.12 | 7.6 ± 8.94 | 5.9 ± 7.95 | 4.43 ± 5.96 | 4.25 ± 5.72 |
| CDV_V_mix_ (mean ± SD) (cm/s) | 0.45 ± 0.1 | 0.4 ± 0.12 | 0.44 ± 0.15 | 0.37 ± 0.14 | 0.36 ± 0.14 | 0.42 ± 0.12 | 0.37 ± 0.13 | 0.41 ± 0.28 | 0.34 ± 0.24 | 0.33 ± 0.24 |
| CDV_FI_mix_ (mean ± SD) (cm/s) | 0.09 ± 0.06 | 0.06 ± 0.06 | 0.04 ± 0.05 | 0.03 ± 0.04 | 0.03 ± 0.04 | 0.06 ± 0.06 | 0.03 ± 0.03 | 0.03 ± 0.03 | 0.02 ± 0.02 | 0.02 ± 0.02 |
| CDV_FI_red_ (mean ± SD) (cm/s) | 0.11 ± 0.11 | 0.08 ± 0.12 | 0.06 ± 0.1 | 0.04 ± 0.07 | 0.04 ± 0.07 | 0.08 ± 0.11 | 0.03 ± 0.04 | 0.03 ± 0.05 | 0.02 ± 0.03 | 0.02 ± 0.03 |
| CDV_FI_blue_ (mean ± SD) (cm/s) | 0.06 ± 0.06 | 0.04 ± 0.04 | 0.03 ± 0.03 | 0.02 ± 0.02 | 0.02 ± 0.02 | 0.04 ± 0.04 | 0.04 ± 0.04 | 0.03 ± 0.03 | 0.02 ± 0.02 | 0.02 ± 0.02 |
| PDI_FI_red_ (mean ± SD) (cm/s) | 0.82 ± 0.37 | 0.5 ± 0.29 | 0.46 ± 0.29 | 0.46 ± 0.31 | 0.47 ± 0.32 | 0.73 ± 0.51 | 0.45 ± 0.25 | 0.48 ± 0.25 | 0.49 ± 0.27 | 0.5 ± 0.28 |

**Legend.** 6M: 6 months; 3Y: 3 years; BL: baseline; CDV: color doppler velocity; FI_mix_: flow intensity, calculated as the average between flow intensity red and flow intensity blue; PDI: power doppler intensity; pRI: perfusion relief intensity; rhPDGF: recombinant human platelet-derived growth factor-BB; SD: standard deviation; V_mix_: perfusion velocity calculated as the average between perfusion velocity red and perfusion velocity blue. VCMX: volume-stable cross-linked collagen matrix; No statistically significant differences were found between the two groups in terms of ultrasonographic tissue perfusion related parameters over 3 years.

**Results from the regression analysis assessment ultrasonographic tissue perfusion over time**

No differences were found between the test and control group in terms of:

- pRI (p=0.24, -0.02 [-0.06, 0.02])
- CDV_V_mix_ (p=0.59, -0.02 [-0.08, 0.05])
- CDV_FI_mix_ (p=0.12, -0.02 [-0.03, 0.00])
- CDV_FI_red_ (p=0.17, -0.03 [-0.06, 0.01])
- CDV_FI_blue_ (p=0.49, -0.01 [-0.02, 0.01])
- PDI_FI_red_ (p=0.83, 0.02 [-0.12, 0.15])

Outcomes from the regression analysis are displayed with their respective p-value, effect estimate and 95% confidence interval.

**Supplementary Table 4**. Clinical and ultrasonographic outcomes at baseline, 6 months, and 3 years at the interventional and untreated contralateral sites.

| **Outcome** | **VCMX + saline**  **(12 patients, 37 sites)** | | | **VCMX + rhPDGF**  **(14 patients, 43 sites)** | | | **Untreated contralateral sites**  **(26 patients, 80 sites)** | | |
| --- | --- | --- | --- | --- | --- | --- | --- | --- | --- |
|  | BL | 6M | 3Y | BL | 6M | 3Y | BL | 6M | 3Y |
| Rec depth  (mean ± SD) (mm) | 3.16 ± 1.26 | 0.70 ± 0.45 | 0.93 ± 0.66 ^a^ | 2.88 ± 0.80 | 0.33 ± 0.50 | 0.50 ± 0.55 ^a^ | 0.89 ± 0.54 | 1.06 ± 0.51 | 1.51 ± 0.93 ^a^ |
| KTW  (mean ± SD) (mm) | 2.18 ± 1.33 | 2.38 ± 1.02 | 2.80 ± 1.02 ^a,c^ | 2.44 ± 0.87 | 2.81 ± 0.85 | 3.17 ± 0.87 ^a,c^ | 2.70 ± 1.09 | 2.81 ± 1.07 | 3.03 ± 1.24 ^a,c^ |
| GT  (mean ± SD) (mm) | 0.91 ± 0.24 | 1.36 ± 0.30 | 1.29 ± 0.42 ^a^ | 0.95 ± 0.29 | 1.65 ± 0.33 | 1.49 ± 0.47 ^a^ | 1.09 ± 0.52 | 1.06 ± 0.55 | 1.05 ± 0.57 ^a^ |
| BBD  (mean ± SD) (mm) | 6.57 ± 3.97 | 6.67 ± 3.42 | 6.93 ±  4.78 ^b^ | 6.26 ± 3.91 | 6.22 ± 3.01 | 6.29 ± 3.54 ^b^ | 4.01 ± 2.67 | 4.63 ± 2.98 | 5.08 ± 3.59 ^b^ |
| EI  (mean ± SD) (GL) | 120.9 ± 44.7 | 144.2 ± 69.4 | 154.9 ± 64.6 ^a^ | 117.6 ± 74.2 | 146.9 ± 81.7 | 162.2 ± 73.8 ^a^ | 124.9 ± 63.3 | 122.7 ± 58.5 | 105.6 ± 57.2 |

**Legend**. 6M: 6 months; 3Y: 3 years; BBD: buccal bone dehiscence/distance from the cemento-enamel junction; BL: baseline; VCMX: cross-linked collagen matrix; EI: mean pixel/echointensity; GL: gray levels; GT: gingival thickness, assessed using ultrasonography 1.5 mm apical to the gingival margin; KTW: keratinized tissue width; n: number; Rec: recession; rhPDGF: recombinant human platelet-derived growth factor-BB; SD: standard deviation; VCMX: volume-stable cross-linked collagen matrix.

^a^ Statistically significant difference favoring treated sites over untreated contralateral sites over 3 years.

^b^ No statistically significant differences were observed between treated sites and untreated contralateral sites over 3 years.

^c^ Statistically significant intra-group difference from 6 month to 3 year

**Supplementary Table 5**. Ultrasonographic tissue elasticity outcomes

| **Group** | **Strain ratio cST/Cr (mean ± SD)** | | | |
| --- | --- | --- | --- | --- |
|  | Baseline | 6 months | 2 years | 3 years |
| **VCMX + saline**  **(12 patients, 37 sites)** | 0.25 ± 0.13 | 0.41 ± 0.21 | 0.52 ± 0.31 | 0.52 ± 0.29 ^a, b, c^ |
| **VCMX + rhPDGF**  **(14 patients, 43 sites)** | 0.23 ± 0.11 | 0.50 ± 0.23 | 0.61 ± 0.37 | 0.63 ± 0.38 ^a, b, c^ |
| **Untreated contralateral sites**  **(26 patients, 80 sites)** | 0.22 ± 0.14 | 0.22 ± 0.16 | 0.23 ± 0.15 | 0.21 ± 0.15 ^a^ |

**Legend**. rhPDGF: recombinant human platelet-derived growth factor-BB; SD: standard deviation; SR: strain ratio; SR was computed as the ratio between Strain_Cr_ and Strain_cST_. SR values < 1 indicate that the coronal soft tissue portion (cST) has higher strain/elasticity than the crown (Cr). VCMX: volume-stable cross-linked collagen matrix.

^a^ Statistically significant difference favoring treated sites over untreated contralateral sites over 3 years.

^b^ Statistically significant increase in strain ratio cST/Cr over 3 years compared to baseline.

^c^ No significant differences between VCMX + saline and VCMX + rhPDGF in terms of strain ratio cST/Cr over time.

**Supplementary Table 6**. Patient-reported outcome measures (PROMs) assessed at baseline, 6 months, and 3 years.

| **PROM** | **VCMX + saline**  **(12 patients, 37 sites)** | | | **VCMX + rhPDGF**  **(14 patients, 43 sites)** | | | **Untreated contralateral sites**  **(26 patients, 80 sites)** | | |
| --- | --- | --- | --- | --- | --- | --- | --- | --- | --- |
|  | BL | 6M | 3Y | BL | 6M | 3Y | BL | 6M | 3Y |
| EST (mean ± SD)  (VAS point 0-100) | 34.0 ± 19.6 | 93.2 ± 8.7 | 86.7 ± 12.6 ^a,b^ | 28.2 ± 12.9 | 90.6 ± 8.1 | 89.1 ± 11.0 ^a,b^ | 66.4 ± 32.9 | 61.8 ± 31.9 | 53.6 ± 22.7 ^a^ |
| DH (mean ± SD)  (VAS point 0-100) | 36.9 ± 21.8 | 8.8 ± 8.4 | 13.4 ± 10.7 ^a,b^ | 31.7 ± 19.9 | 6.7 ± 7.0 | 9.3 ± 10.4 ^a,b^ | 14.6 ± 16.3 | 12.5 ± 16.1 | 31.3 ± 21.3 ^a,d^ |
| SAT (mean ± SD)  (VAS point 0-100) | / | 87.2 ± 10.5 | 86.7 ± 14.4 ^c^ | / | 92.3 ± 10.9 | 94.2 ± 6.4 ^c^ | / | / | / |
| Remembered post-surgical morbidity (mean ± SD)  (VAS point 0-100) | / | / | 20.3 ± 11.7 ^b^ | / | / | 17.9 ± 12.2 ^b^ | / | / | / |
| Subjects that would be willing to undergo the same procedure again (N, %) | / | / | 11, 91.7 ^b^ | / | / | 13, 92.9 ^b^ | / | / | / |
| Subjects that would recommend the same procedure to friends and family (N, %) | / | / | 10, 83.3 ^b^ | / | / | 13, 92.9 ^b^ | / | / | / |

**Legend**. 6M: 6 months; 3Y: 3 years; BL: baseline; DH: patient-reported dental hypersensitivity; EST: patient-reported esthetic assessment; N: number; rhPDGF: recombinant human platelet-derived growth factor-BB; SAT: patient-reported treatment satisfaction; SD: standard deviation; VAS: visual analogue scale; VCMX: volume-stable cross-linked collagen matrix.

^a^ Statistically significant difference favoring treated sites over untreated contralateral sites over 3 years

^b^ No statistically significant differences were noticed between the test and control group for this outcome.

^c^ Significant differences favoring the test group over the control group were noticed for this outcome over the 3 years.

^d^ Statistically significant intra-group difference from 6 month to 3 year

**Supplementary Table 7**. Gingival phenotype assessment at different time points using color-coded probes.

| **Outcome** | **VCMX + saline**  **(12 patients, 37 sites)** | | | **VCMX + rhPDGF**  **(14 patients, 43 sites)** | | | **Untreated contralateral sites**  **(26 patients, 80 sites)** | | |
| --- | --- | --- | --- | --- | --- | --- | --- | --- | --- |
|  | BL | 6M | 3Y | BL | 6M | 3Y | BL | 6M | 3Y |
| Sites with thin phenotype (n, %) | 22, 59.5 | 0, 0 | 0, 0 | 23, 53.5 | 0, 0 | 0, 0 | 49, 61.3 | 48, 60 | 48, 60 |
| Sites with medium phenotype (n, %) | 11, 29.7 | 5, 13.5 | 5, 13.5 | 14, 32.6 | 2, 4.7 | 3, 7.0 | 21, 26.3 | 23, 28.9 | 24, 30 |
| Sites with thick phenotype (n, %) | 4, 10.8 | 18, 48.6 | 21, 56.8 | 6, 14.0 | 4, 9.3 | 10, 23.3 | 9, 11.3 | 8, 10 | 8, 10 |
| Sites with very thick phenotype (n, %) | 0, 0 | 14, 37.8 | 11, 29.7 | 0, 0 | 37, 86.0 | 30, 69.8 | 1, 1.3 | 1, 1.3 | 0, 0 |

**Legend**. 3Y: 3 years; 6M: 6 months; BL: baseline; n: number; rhPDGF: recombinant human platelet-derived growth factor-BB; VCMX: cross-linked collagen matrix. No statistically significant changes in outcomes were observed within each group, nor among the groups, from 6 months to 3 years.

**Supplementary Fig. 1.** Ultrasonographic dynamic tissue perfusion evaluation over 3 years displayed as perfusion relief intensity (pRI). pRI refers to the number of pixel within the region of interest (ROI, either the graft or the flap) that relate to the intensity of the respective value. Areas with zero intensity/perfusion within the ROI are shown in black, while areas with half of the maximum intensity/perfusion and maximum intensity/perfusion are visualized in purple and red, respectively.


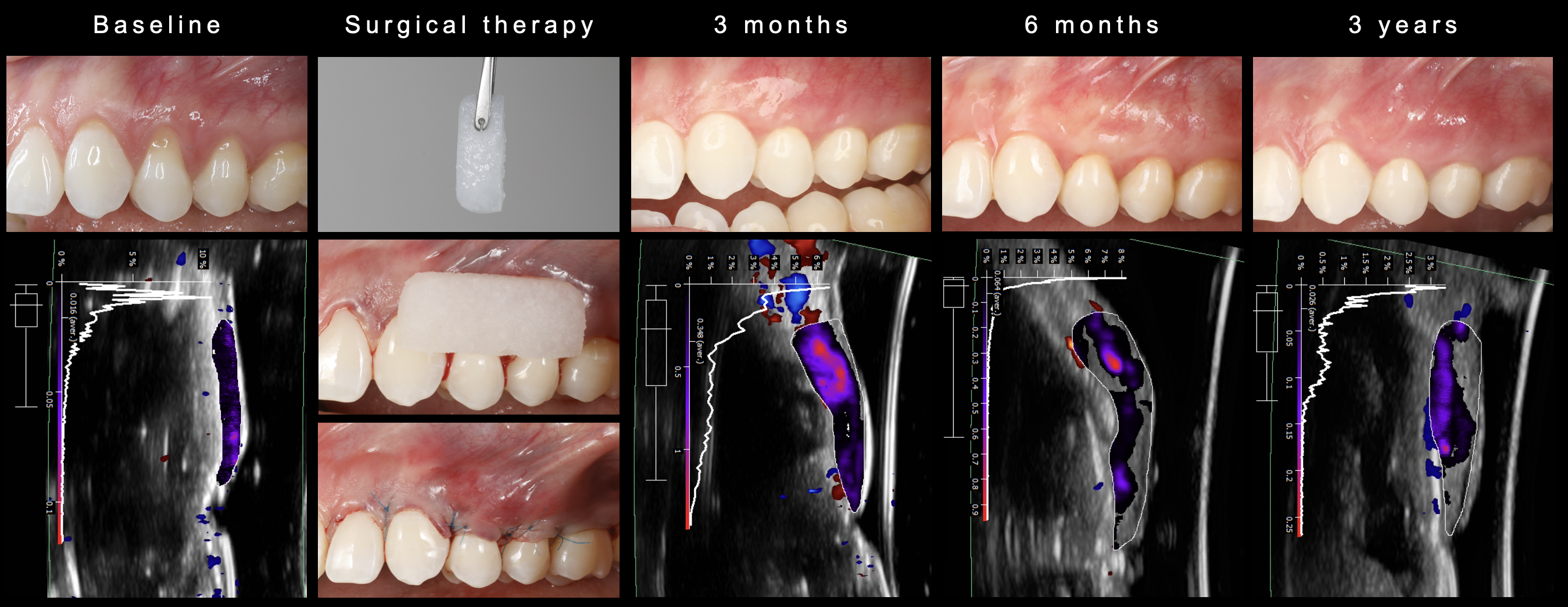


REFERENCES

Barootchi, S., Tavelli, L., Majzoub, J., Chan, H. L., Wang, H. L., & Kripfgans, O. D. (2022). Ultrasonographic Tissue Perfusion in Peri-implant Health and Disease. *J Dent Res, 101*(3), 278-285. doi:10.1177/00220345211035684

Borges, T., Fernandes, D., Almeida, B., Pereira, M., Martins, D., Azevedo, L., & Marques, T. (2020). Correlation between alveolar bone morphology and volumetric dimensional changes in immediate maxillary implant placement: A 1-year prospective cohort study. *J Periodontol, 91*(9), 1167-1176. doi:10.1002/JPER.19-0606

Cairo, F., Cortellini, P., Pilloni, A., Nieri, M., Cincinelli, S., Amunni, F., . . . Tonetti, M. S. (2016). Clinical efficacy of coronally advanced flap with or without connective tissue graft for the treatment of multiple adjacent gingival recessions in the aesthetic area: a randomized controlled clinical trial. *J Clin Periodontol, 43*(10), 849-856. doi:10.1111/jcpe.12590

Cairo, F., Nieri, M., Cincinelli, S., Mervelt, J., & Pagliaro, U. (2011). The interproximal clinical attachment level to classify gingival recessions and predict root coverage outcomes: an explorative and reliability study. *J Clin Periodontol, 38*(7), 661-666. doi:10.1111/j.1600-051X.2011.01732.x

Cairo, F., Rotundo, R., Miller, P. D., & Pini Prato, G. P. (2009). Root coverage esthetic score: a system to evaluate the esthetic outcome of the treatment of gingival recession through evaluation of clinical cases. *J Periodontol, 80*(4), 705-710. doi:10.1902/jop.2009.080565

Chan, H. L., & Kripfgans, O. D. (2020a). Ultrasonography for diagnosis of peri-implant diseases and conditions: a detailed scanning protocol and case demonstration. *Dentomaxillofac Radiol, 49*(7), 20190445. doi:10.1259/dmfr.20190445

Chan, H. L., & Kripfgans, O. D. (2020b). Ultrasonography for diagnosis of peri-implant diseases and conditions: a detailed scanning protocol and case demonstration. *Dentomaxillofac Radiol*, 20190445. doi:10.1259/dmfr.20190445

Chan, H. L., Sinjab, K., Chung, M. P., Chiang, Y. C., Wang, H. L., Giannobile, W. V., & Kripfgans, O. D. (2017). Non-invasive evaluation of facial crestal bone with ultrasonography. *PLoS One, 12*(2), e0171237. doi:10.1371/journal.pone.0171237

Choi, Y. J., Lee, J. H., & Baek, J. H. (2015). Ultrasound elastography for evaluation of cervical lymph nodes. *Ultrasonography, 34*(3), 157-164. doi:10.14366/usg.15007

Dyrla, P., Gil, J., Kosik, K., Schneditz, D., Saracyn, M., Niemczyk, S., & Lubas, A. (2019). Doppler tissue perfusion measurement is a sensitive and specific tool for a differentiation between malignant and inflammatory pancreatic tumors. *PLoS One, 14*(4), e0215944. doi:10.1371/journal.pone.0215944

Fons-Badal, C., Alonso Perez-Barquero, J., Martinez-Martinez, N., Faus-Lopez, J., Fons-Font, A., & Agustin-Panadero, R. (2020). A novel, fully digital approach to quantifying volume gain after soft tissue graft surgery. A pilot study. *J Clin Periodontol, 47*(5), 614-620. doi:10.1111/jcpe.13235

Galarraga-Vinueza, M. E., Barootchi, S., Mancini, L., Sabri, H., Schwarz, F., Gallucci, G. O., & Tavelli, L. (2024). Echo-intensity characterization at implant sites and novel diagnostic ultrasonographic markers for peri-implantitis. *J Clin Periodontol*. doi:10.1111/jcpe.13976

Gennisson, J. L., Deffieux, T., Fink, M., & Tanter, M. (2013). Ultrasound elastography: principles and techniques. *Diagn Interv Imaging, 94*(5), 487-495. doi:10.1016/j.diii.2013.01.022

Kamal, E. F., Kamal, R. M., Mahmoud, A. M., Mekhaimar, M. I., & Hanafy, M. M. (2022). Comparative study between conventional ultrasound strain elastography

and an AI-enabled elastography software in differentiating breast masses. *Egypt J Radiol Nucl Med*(53), 33.

Kim, S. Y., Kim, E. K., Moon, H. J., Yoon, J. H., & Kwak, J. Y. (2015). Application of Texture Analysis in the Differential Diagnosis of Benign and Malignant Thyroid Nodules: Comparison With Gray-Scale Ultrasound and Elastography. *AJR Am J Roentgenol, 205*(3), W343-351. doi:10.2214/AJR.14.13825

Mahmoud, A. M., & Ali, M. T. M. (2020). U. S. P. a. T. Office.

Mancini, L., Khehra, A., Nguyen, T., Barootchi, S., & Tavelli, L. (2023). Echo intensity and gray-level co-occurrence matrix analysis of soft tissue grafting biomaterials and dental implants: an in vitro ultrasonographic pilot study. *Dentomaxillofac Radiol*, 20230033. doi:10.1259/dmfr.20230033

Mascardo, K. C., Tomack, J., Chen, C. Y., Mancini, L., Kim, D. M., Friedland, B., . . . Tavelli, L. (2024). Risk indicators for gingival recession in the esthetic zone: A cross-sectional clinical, tomographic, and ultrasonographic study. *J Periodontol, 95*(5), 432-443. doi:10.1002/JPER.23-0357

Parvini, P., Galarraga-Vinueza, M. E., Obreja, K., Magini, R. S., Sader, R., & Schwarz, F. (2021). Prospective study assessing three-dimensional changes of mucosal healing following soft tissue augmentation using free gingival grafts. *J Periodontol, 92*(3), 400-408. doi:10.1002/JPER.19-0640

Rosenbaum, C., Wach, S., Kunath, F., Wullich, B., Scholbach, T., & Engehausen, D. G. (2013). Dynamic tissue perfusion measurement: a new tool for characterizing renal perfusion in renal cell carcinoma patients. *Urol Int, 90*(1), 87-94. doi:10.1159/000341262

Rubins, R. P., Tolmie, P. N., Corsig, K. T., Kerr, E. N., & Kim, D. M. (2013). Subepithelial connective tissue graft with growth factor for the treatment of maxillary gingival recession defects. *Int J Periodontics Restorative Dent, 33*(1), 43-50.

Rubins, R. P., Tolmie, P. N., Corsig, K. T., Kerr, E. N., & Kim, D. M. (2014). Subepithelial connective tissue graft with purified rhPDGF-BB for the treatment of mandibular recession defects: a consecutive case series. *Int J Periodontics Restorative Dent, 34*(3), 315-321. doi:10.11607/prd.1635

Schmitt, C. M., Matta, R. E., Moest, T., Humann, J., Gammel, L., Neukam, F. W., & Schlegel, K. A. (2016). Soft tissue volume alterations after connective tissue grafting at teeth: the subepithelial autologous connective tissue graft versus a porcine collagen matrix - a pre-clinical volumetric analysis. *J Clin Periodontol, 43*(7), 609-617. doi:10.1111/jcpe.12547

Scholbach, T., Girelli, E., & Scholbach, J. (2005). Dynamic tissue perfusion measurement: a novel tool in follow-up of renal transplants. *Transplantation, 79*(12), 1711-1716. doi:10.1097/01.tp.0000164145.89275.02

Scholbach, T., & Scholbach, J. (2009). Dynamic Sonographic Tissue Perfuson Measurement. *J Med Ultrasound, 17*(2), 71-85.

Sigrist, R. M. S., Liau, J., Kaffas, A. E., Chammas, M. C., & Willmann, J. K. (2017). Ultrasound Elastography: Review of Techniques and Clinical Applications. *Theranostics, 7*(5), 1303-1329. doi:10.7150/thno.18650

Tattan, M., Sinjab, K., Lee, E., Arnett, M., Oh, T. J., Wang, H. L., . . . Kripfgans, O. D. (2019). Ultrasonography for chairside evaluation of periodontal structures: A pilot study. *J Periodontol*. doi:10.1002/JPER.19-0342

Tavelli, L., & Barootchi, S. (2024). Soft tissue elasticity at teeth and implant sites. A novel outcome measure of the soft tissue phenotype. *J Periodontal Res*. doi:10.1111/jre.13296

Tavelli, L., Barootchi, S., Majzoub, J., Chan, H. L., Giannobile, W. V., Wang, H. L., & Kripfgans, O. D. (2021a). Ultrasonographic tissue perfusion analysis at implant and palatal donor sites following soft tissue augmentation: A clinical pilot study. *J Clin Periodontol, 48*(4), 602-614. doi:10.1111/jcpe.13424

Tavelli, L., Barootchi, S., Majzoub, J., Chan, H. L., Giannobile, W. V., Wang, H. L., & Kripfgans, O. D. (2021b). Ultrasonographic tissue perfusion analysis at implant and palatal donor sites following soft tissue augmentation: A clinical pilot study. *J Clin Periodontol*. doi:10.1111/jcpe.13424

Tavelli, L., Barootchi, S., Majzoub, J., Siqueira, R., Mendonca, G., & Wang, H. L. (2021). Volumetric changes at implant sites: A systematic appraisal of traditional methods and optical scanning-based digital technologies. *J Clin Periodontol, 48*(2), 315-334. doi:10.1111/jcpe.13401

Tavelli, L., Barootchi, S., Rodriguez, M. V., Mancini, L., Majzoub, J., Travan, S., . . . Giannobile, W. V. (2022). Recombinant human platelet-derived growth factor improves root coverage of a collagen matrix for multiple adjacent gingival recessions: A triple-blinded, randomized, placebo-controlled trial. *J Clin Periodontol, 49*(11), 1169-1184. doi:10.1111/jcpe.13706

Tavelli, L., Kripfgans, O. D., Chan, H. L., Vera Rodriguez, M., Sabri, H., Mancini, L., . . . Barootchi, S. (2023). Doppler ultrasonographic evaluation of tissue revascularization following connective tissue graft at implant sites. *J Clin Periodontol*. doi:10.1111/jcpe.13889

Tian, J., Wei, D., Zhao, Y., Di, P., Jiang, X., & Lin, Y. (2019). Labial soft tissue contour dynamics following immediate implants and immediate provisionalization of single maxillary incisors: A 1-year prospective study. *Clin Implant Dent Relat Res, 21*(3), 492-502. doi:10.1111/cid.12786

Tonetti, M. S., Cortellini, P., Pellegrini, G., Nieri, M., Bonaccini, D., Allegri, M., . . . Zuhr, O. (2018). Xenogenic collagen matrix or autologous connective tissue graft as adjunct to coronally advanced flaps for coverage of multiple adjacent gingival recession: Randomized trial assessing non-inferiority in root coverage and superiority in oral health-related quality of life. *J Clin Periodontol, 45*(1), 78-88. doi:10.1111/jcpe.12834

Xue, F., Zhang, R., Cai, Y., Zhang, Y., Kang, N., & Luan, Q. (2021). Three-dimensional quantitative measurement of buccal augmented tissue with modified coronally advanced tunnel technique and de-epithelialized gingival graft: a prospective case series. *BMC Oral Health, 21*(1), 157. doi:10.1186/s12903-021-01522-2

Zucchelli, G., & De Sanctis, M. (2000). Treatment of multiple recession-type defects in patients with esthetic demands. *J Periodontol, 71*(9), 1506-1514. doi:10.1902/jop.2000.71.9.1506

Zucchelli, G., Mele, M., Mazzotti, C., Marzadori, M., Montebugnoli, L., & De Sanctis, M. (2009). Coronally advanced flap with and without vertical releasing incisions for the treatment of multiple gingival recessions: a comparative controlled randomized clinical trial. *J Periodontol, 80*(7), 1083-1094. doi:10.1902/jop.2009.090041
